# Overcoming Antigen Escape and T-Cell Exhaustion in CAR-T Therapy for Leukemia

**DOI:** 10.3390/cells13181596

**Published:** 2024-09-23

**Authors:** Elżbieta Bartoszewska, Maciej Tota, Monika Kisielewska, Izabela Skowron, Kamil Sebastianka, Oliwia Stefaniak, Klaudia Molik, Jakub Rubin, Karolina Kraska, Anna Choromańska

**Affiliations:** 1 Faculty of Medicine, Wroclaw Medical University, Mikulicza-Radeckiego 5, 50-345 Wroclaw, Polandmonika.kisielewska@student.umw.edu.pl (M.K.); izabela.skowron@student.umw.edu.pl (I.S.); kamil.sebastianka@student.umw.edu.pl (K.S.); oliwia.stefaniak@student.umw.edu.pl (O.S.); klaudia.molik@student.umw.edu.pl (K.M.); jakub.rubin@student.umw.edu.pl (J.R.); karolina.kraska@student.umw.edu.pl (K.K.); 2 Student Research Group No K148, Faculty of Pharmacy, Wroclaw Medical University, Borowska 211A, 50-556 Wroclaw, Poland; 3 Department of Molecular and Cellular Biology, Wroclaw Medical University, Borowska 211A, 50-556 Wroclaw, Poland

**Keywords:** leukemia, pediatric oncology, CAR-T therapy, acute lymphoblastic leukemia, acute myeloid leukemia, antigen escape, T-cell exhaustion, cytokine release syndrome, immune effector cell-associated neurotoxicity syndrome, dual-targeting CAR-Ts

## Abstract

Leukemia is a prevalent pediatric cancer with significant challenges, particularly in relapsed or refractory cases. Chimeric antigen receptor T-cell (CAR-T) therapy has emerged as a personalized cancer treatment, modifying patients’ T cells to target and destroy resistant cancer cells. This study reviews the current therapeutic options of CAR-T therapy for leukemia, addressing the primary obstacles such as antigen escape and T-cell exhaustion. We explore dual-targeting strategies and their potential to improve treatment outcomes by preventing the loss of target antigens. Additionally, we examine the mechanisms of T-cell exhaustion and strategies to enhance CAR-T persistence and effectiveness. Despite remarkable clinical successes, CAR-T therapy poses risks such as cytokine release syndrome (CRS) and immune effector cell-associated neurotoxicity syndrome (ICANS). Our findings highlight the need for ongoing research to optimize CAR-T applications, reduce toxicities, and extend this innovative therapy to a broader range of hematologic malignancies. This comprehensive review aims to provide valuable insights for improving leukemia treatment and advancing the field of cancer immunotherapy.

## 1. Introduction

### 1.1. Leukemia

Leukemia is the most frequently diagnosed oncologic condition among the pediatric patient population [1]. In 2022, leukemia was the 13th most commonly diagnosed malignancy and the 10th leading cause of cancer-related mortality globally. It was estimated that there were over 487,000 new cases of leukemia and approximately 305,000 deaths resulting from the disease [2]. Although overall survival rates for acute lymphoblastic leukemia (ALL) and acute myeloid leukemia (AML), through the implementation of risk-stratified chemotherapy regimens, have exceeded 90% and 70%, respectively, children who experience relapsed or refractory disease still face unfavorable prognoses [3,4,5]. Regardless of substantial advancements in the treatment of these disorders, their etiology remains elusive. A broad and varied array of genetic and environmental factors has been suggested. Researchers have examined the influence of numerous factors, including inherited and acquired genetic mutations and exposure to radiation and various chemicals during preconception, pregnancy, and throughout an individual’s life. The potential impact of inherited genetic variations and disorders, pre-existing medical conditions, infectious agents, occupational exposures, previous treatments, and a multitude of other factors have been considered. However, no single factor has been universally identified as applicable to all instances [6,7].

Acute leukemias are malignant clonal disorders affecting the blood-forming organs, involving one or more cell lines within the hematopoietic system. These disorders are characterized by the widespread replacement of bone marrow with abnormal, immature, and undifferentiated hematopoietic cells. This replacement results in decreased erythrocytes and platelets in the peripheral blood. The classification of these disorders is based on the origin of the abnormal hematopoietic cells, which may be lymphoid, myeloid, mixed, or undifferentiated. Conversely, chronic leukemias represent a wide range of diseases marked by the uncontrolled proliferation and expansion of mature, differentiated cells within the hematopoietic system. Consequently, chronic leukemias are classified according to the type of hematopoietic cells involved [6,8].

### 1.2. Chimeric Antigen Teceptor T-Cell (CAR-T) Therapy 

**Figure 1:**
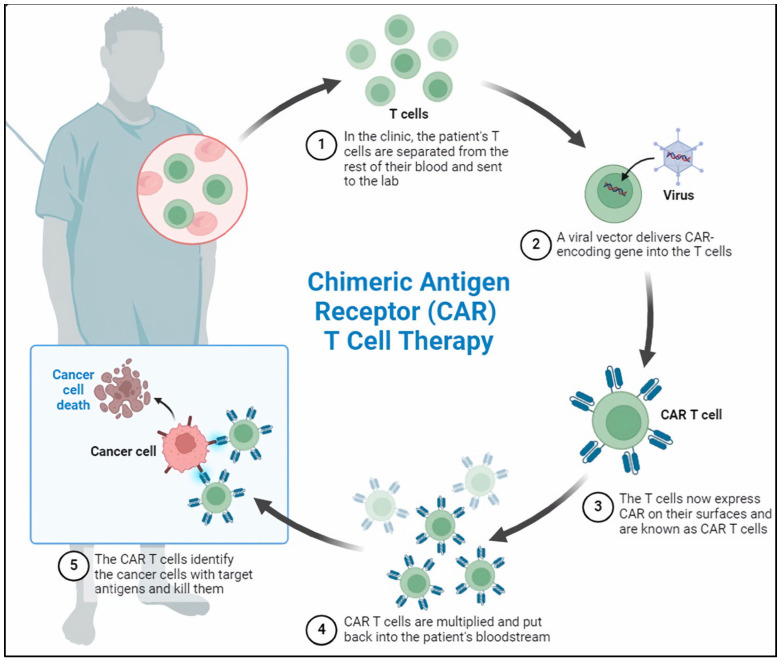
Chimeric antigen receptor T-cell (CAR-T) therapy. CAR-T therapy involves a multi-step process that begins with extracting T cells from the patient’s peripheral blood. These T cells are genetically engineered using a viral vector to express chimeric antigen receptors (CARs). Following genetic modification, the T cells are expanded ex vivo, meaning they are cultivated and multiplied outside the body under controlled laboratory conditions. Once a sufficient number of modified T cells are obtained, they are reinfused into the patient to serve as a therapeutic intervention.

Chimeric antigen receptor T-cell (CAR-T) therapy constitutes a significant innovation in personalized cancer treatment. This therapeutic approach involves the genetic modification of a patient’s T cells to express a synthetic receptor capable of binding to a specific tumor antigen [Figure 1]. Subsequently, these CAR-Ts are expanded ex vivo for clinical application and reintroduced into the patient’s body to target and eradicate chemotherapy-resistant cancer cells. The implementation of CAR-T therapy, particularly in the treatment of B-cell malignancies, has yielded remarkable clinical outcomes, including high rates of complete remission. In 2017, the Food and Drug Administration (FDA) approved the initial CAR-T therapies. These therapies were explicitly approved for the treatment of relapsed or refractory diffuse large B-cell lymphoma (DLBCL) and for the treatment of patients up to 25 years of age who have ALL that is either refractory or in a second or subsequent relapse [9,10]. Although this therapy is associated with potentially life-threatening toxicities, including cytokine release syndrome (CRS) and immune effector cell-associated neurotoxicity syndrome (ICANS), the advantages of CAR-T therapy significantly outweigh these risks [11,12].

The foremost challenges and inquiries for clinical researchers revolve around the optimal utilization of CAR-Ts and the strategies to surmount resistance mechanisms to these cells. A principal clinical query pertains to the timing and the necessity of allogeneic hematopoietic stem cell transplantation (allo-HSCT). Regarding resistance, it is established that the loss of target antigens, notably CD19, significantly contributes to resistance [13]. Nevertheless, current research exploring alternative targets, such as CD22, and CAR-Ts expressing dual-targeting antigen receptors has shown promising initial outcomes. These findings engender substantial optimism that the efficacy and broader application of CAR-T therapy in B-cell acute lymphoblastic leukemia (B-ALL) will progressively improve [14]. Conversely, the optimism is less pronounced, and the challenges are more formidable, concerning the application of CAR-Ts in T-cell leukemias and acute myeloid leukemia, due to the relative scarcity of suitable leukemia surface targets that are not also present in normal hematopoietic progenitors [15].

### 1.3. Major Molecular Challenges—Antigen Escape and T-Cell Exhaustion

Despite its remarkable success, CAR-T therapy encounters substantial molecular challenges, including antigen escape and the phenomenon of T-cell exhaustion.

#### 1.3.1. Antigen Escape

One significant challenge of CAR-T therapy is the development of tumor resistance to single antigen-targeted CAR constructs. Initially, CAR-Ts targeting a single antigen can achieve high response rates; however, many patients experience partial or complete loss of target antigen expression in malignant cells, a phenomenon known as antigen escape [16]. For instance, while 70–90% of relapsed and/or refractory ALL patients show durable responses to CD19-targeted CAR-T therapy, recent data indicate that 30–70% of these patients develop disease resistance due to CD19 antigen downregulation or loss [17,18]. Similarly, B-cell maturation antigen (BCMA) expression downregulation or loss has been observed in multiple myeloma patients treated with BCMA-targeted CAR-Ts [18,19,20].

To reduce relapse rates in CAR-T treatment of both hematological malignancies and solid tumors, strategies now often involve targeting multiple antigens [21]. These strategies result in prolonged durable remission rates. Several clinical trials investigate combinations such as CD19 and CD20 or CD19 and CD22 [22,23,24]. Preliminary results from trials using dual-targeted CAR-Ts (CD19/CD22 or CD19/BCMA) have shown promising outcomes, particularly in adult patients with ALL and diffuse large B-cell lymphoma [23,24]. Additionally, BCMA/CD19-targeted CARs have demonstrated high efficacy and favorable safety profiles in multiple myeloma treatment [22].

#### 1.3.2. T-Cell Exhaustion

The efficacy of CAR-Ts in vivo is determined by their functional state. Exhausted CAR-Ts demonstrate a reduced capacity for proliferation, diminished anti-tumor activity, and decreased persistence [25]. CAR-T exhaustion can arise from prolonged antigen stimulation and has been identified as a critical factor contributing to nonresponse and relapse following CAR-T therapy [26]. Elevated concentrations of lymphocyte-activation gene-3 (LAG-3) and T-cell immunoglobulin and mucin domain-containing protein 3 (TIM-3) in CAR-T infusion samples are correlated with a diminished overall response rate (ORR) of CART19 cell therapy and a heightened probability of early relapse of DLBCL [27]. Furthermore, an increase in T cells that express LAG-3, along with a decreased ability to release cytokines upon activation, can undermine the effectiveness of CART19 therapy, leading to a relapse with CD19-positive cells [28].

Nevertheless, the triggers and mechanisms underlying CAR-T exhaustion are still poorly understood and appear complex. Consequently, it is imperative to elucidate the regulatory network governing CAR-T exhaustion and to investigate effective solutions.

### 1.4. Aim of the Study

Our objective was to comprehensively review the current status of therapeutic CAR-T options for pediatric and adult patients diagnosed with leukemia. Additionally, we sought to identify the treatment challenges associated with the manufacturing process and the toxicities that may arise from such therapies.

## 2. Materials and Methods

There is a great number of studies that provide solutions for problems described in this paper. Electronic searches were conducted to find them on sites such as PubMed, Google Scholar, and ScienceDirect. The analyzed articles are also available in Web of Science and the Scopus databases. The data were selected based on keywords included in this article. 

## 3. Antigen Escape in CAR-T Therapy

### 3.1. Mechanisms of Antigen Escape

Tumor cell features, such as the content of the outer cell membrane, are a significant predictor of CAR-T treatment susceptibility and clinical outcome [29]. The function of T cells can be navigated to a particular tumor-associated antigen by expressing CAR. Those particles are made up of an antigen recognition domain, often a single-chain variable immunoglobulin fragment, connected to T-cell activation (CD3ζ) and co-stimulation (typically CD28 or 4-1BB) of intracellular signaling domains. When an antigen is recognized, the T cell activates, beginning proliferation and cytokines secretion, before immediately lysing the targeted cell. The research has revealed that aiming at neoplastic cells with CD19-specific CARs has great potential, especially in the treatment of B-cell acute lymphoblastic leukemia [30]. This mechanism shows the importance of cancerous cells’ antigens and explains how antigen escape plays a major role in CAR-T treatment resistance and leukemia relapse [31].

**Figure 2:**
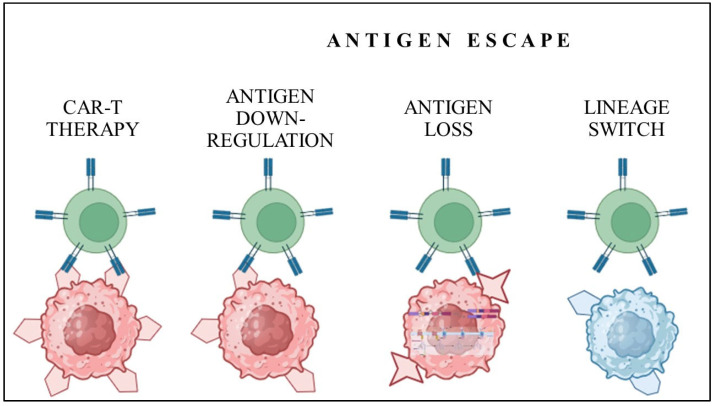
Mechanisms of antigen escape resulting in CAR-T therapy ineffectiveness.

Antigen escape refers to the process in which tumor cells evade immune detection and destruction by altering or losing the specific antigens that targeted therapies, such as CAR-T therapy, are designed to recognize. This phenomenon allows tumor cells to survive and proliferate despite the presence of leukocytes engineered to target and eliminate them. The main pathways of antigen escape comprise three mechanisms [Figure 2]. One comprises downregulation of the surfaces’ targeted antigen to the level insufficient for CAR-T activation. Another is based on molecular changes in cell membranes’ crucial proteins, leading to a lack of epitopes being recognized by CAR-T. The last one occurs on a genetic level, and it is based on a lineage switch [32].

Tumor cells can reduce the expression levels of antigens that are being targeted by CAR-T to avoid immune surveillance. This downregulation can occur through various mechanisms—including epigenetic modifications such as DNA methylation and histone alteration that silence gene expression or post-transcriptional modifications—that employ microRNAs and RNA-binding proteins that affect protein stability and localization. Post-translational modifications, such as ubiquitination and glycosylation, also play a part in this process. For example, the downregulation of CD19 has been observed in some patients who relapse after initially responding to CD19-targeted CAR-T therapy [33,34,35]. 

Loss of the target antigens is based on the complete loss of the targeted antigen on the surface of tumor cells. For instance, in CAR-T therapies targeting CD19, such as those used for B-cell malignancies, some tumor cells may undergo genetic alterations that result in the deletion or mutation of the CD19 gene, leading to the absence of the CD19 protein on their surface. These CD19-negative cells can then evade detection and destruction by CAR-Ts. Moreover, after attaining remission with CD19 CARs, individuals may relapse with a comparable illness that lacks surface expression of a CD19 molecule that may bind anti-CD19 antibodies [32]. 

Another challenge is the changes in glycosylation patterns of the target antigen and splicing variants. Recent studies indicate that the expression of the Golgi-resident intramembrane protease signal peptide peptidase-like 3 (SPPL3) in malignant B cells is a significant CAR treatment resistance regulator. Loss of SPPL3 causes CD19 hyperglycosylation, which directly reduces CAR-T effectiveness and anti-tumor cytotoxicity. On the other hand, the over-expression of SPPL3 promotes CD19 protein loss, permitting therapy resistance. Alternative splicing of produced CD19 messenger RNA can remove domains required for the integration of the membrane or the CAR binding epitope, which leads to loss of surface expression or areas required for CAR–antigen interaction [36]. 

Alternative splicing mostly involves deletions throughout the CD19 locus, as well as frameshift de novo and missense mutations in exon 2 of CD19 in certain relapse cases. Pull-down/siRNA experiments revealed SRSF3 as a splicing regulator implicated in the retention of exon 2, with lower levels in relapsed B-ALL. Exon 2 skipping permits the production of the N-terminally shortened CD19 variant, causing a lack of CAR-T activation. Therefore, this resistance mechanism relies on a combination comprising detrimental mutations and subsequent selection for alternatively spliced RNA isoforms [37].

Lineage switch occurs when a patient relapses with a genetically related but phenotypically different malignancy. It is most often seen in patients who harbor mixed lineage leukemia (MLL) rearrangements, such as infants with B-ALL, when the leukemic phenotype changes from lymphoid to myeloid in response to CD19-directed immunotherapy. The evolved leukemic population not only no longer expresses CD19 but also acquires other phenotypic characteristics of AML [32].

### 3.2. Role of Tumor Heterogeneity and Clonal Evolution

Neoplasms consist of a diverse population of cells with various genetic and phenotypic profiles. This variety provides a base for the selection of clones that can evade immune detection. Moreover, neoplastic cells often exhibit genetic instability, which contributes to the rapid evolution and adaptation of cancer cells. This fluctuation can lead to the emergence of new mutations or epigenetic changes that result in the loss or modification of the target antigen. Tumor heterogeneity and clonal evolution are key factors that contribute to antigen escape [38].

Tumor heterogeneity is displayed as the presence of different subclones within a tumor. It means that the targeted antigen is not expressed on the same level in every cell. Some subclones may naturally express lower levels of the target antigen or have alternative mechanisms for survival that do not rely on the antigen being targeted by CAR-Ts. This intratumoral diversity can lead to the survival of neoplastic cells and cause treatment resistance or remission [39].

The other important aspect, clonal evolution, is the outcome of selective pressure caused by CAR-T therapy and drives the evolution of tumor cells. Cells which are able to evade immune detection by losing or downregulating the target antigen have a survival advantage and have a better chance of proliferating. This process can lead to a relapse with a tumor that is predominantly composed of antigen-negative or antigen-low cells [40]. 

### 3.3. Impact of Antigen Escape on Therapy Outcomes and Relapse Rates

The phenomenon of antigen escape has significant implications for the outcomes of CAR-T therapy. Moreover, it is one of the primary mechanisms leading to relapse in patients treated with CAR-Ts. The effectiveness of CAR-T therapy relies on the ability of the engineered T cells to recognize and eliminate tumor cells expressing the target antigen. When tumor cells lose or downregulate the target antigen, the efficacy of the therapy is compromised. Studies have shown that patients who relapse after CAR-T therapy often have tumors that no longer express the target antigen. This antigen escape is a major barrier to achieving long-term remissions in patients undergoing CAR-T therapy [41].

Relapses can be categorized based on their place of origin as either bone marrow, extramedullary, or mixed. The rate of disease recurrence due to antigen escape varies depending on the type of cancer and the target antigen. In patients treated for B-ALL, regression due to CD19-negative clones is a well-known phenomenon [42]. Moreover, a significant number of patients relapse following relapsed/refractory B-cell CAR-T treatment [43]. It has also been documented that nearly all multiple myeloma (MM) patients experience disease recurrence [16]. Furthermore, around 10% of individuals diagnosed with DLBCL fail to respond to treatment, and up to one-third of those in full remission ultimately relapse [44]. These relapses pose a significant challenge as they often require alternative therapeutic strategies, such as the development of CAR-Ts targeting different antigens or combination therapies.

## 4. Molecular Basis of T-Cell Exhaustion

T-cell exhaustion was primarily observed in chronic viral infections such as HIV or hepatitis C. However, it is also present in cancers; the extended antigen exposure and the immunosuppressive microenvironment of the tumor result in T-cell exhaustion. A loss of effector function, expression of inhibitory receptors, and limited proliferative capability characterize T-cell exhaustion [45,46,47]. 

One of the more well-known signaling pathways contributing to T-cell exhaustion is the PD-1/PD-L1 pathway. Programmed death-1 (PD-1) is an inhibitory receptor that is upregulated on the exhausted cells and interacts with its ligand (PD-L1). As a result, T cells exhibit insufficient cytotoxic activity towards cancerous cells [48]. Another receptor that takes part in T-cell exhaustion is cytotoxic T-lymphocyte-associated protein 4 (CTLA-4), which competes with CD28 in binding to CD80/CD86 on APC, thus inhibiting T-cell stimulation [49,50]. T-cell immunoglobulin mucin-3 (TIM-3) also participates in the exhaustion process. TIM-3’s task is to bind with its ligand galectin-9, which results in T-cell death. Overexpressed TIM-3 on the cells leads to suppressed production of cytokines and limited proliferation [51,52]. Similarly, lymphocyte-activation gene 3 (LAG-3) negatively regulated the activation of T cells by binding MHC class II, contributing to the exhausted phenotype in T cells [53].

Studies have shown that epigenetic changes in T cells also play a key role in the process of exhaustion. One of the epigenetic changes in T cells is the increase of chromatin accessibility in the regions associated with exhaustion genes, such as *Havcr2* and *Pdcd* [54]. These differences result in the occurrence of approximately 6000 gene loci which are expressed differently when compared to non-exhausted cells [55,56,57]. Another way of epigenetic alteration is de novo DNA methylation performed by DNMT3A demethylase. DNA methylation occurs at *Tcf7*, *Ifng*, and *Myc* loci, reducing cell proliferation, dysfunction, and general exhaustion [58]. 

Taking everything into account, due to T-cell exhaustion caused by prolonged antigen exposure, upregulation of inhibitory receptors such as PD-1 and CTLA-4, and epigenetic modifications, CAR-T therapy in leukemia may be ineffective [Table 1]. Patients with the exhausted phenotype of CAR-Ts are more prone to have lower response rates and shorter durations of remissions. That is why it is crucial to examine the underlying causes of the exhaustion in order to target them and increase the efficacy of CAR-T therapy [59].

## 5. Innovative Strategies to Combat Antigen Escape

### 5.1. Development of Multi-Targeted CAR-Ts

Multi-targeted CAR-T therapy is an innovative method aimed at improving the efficiency of cancer treatment by targeting multiple antigens simultaneously. It provides prevention from antigen loss and tumor heterogeneity, which are important and common factors in treatment resistance. The development of multi-targeted CAR-Ts, including pooled, dual, and tandem CARs, represents a significant advancement in cancer immunotherapy that is used to overcome the limitations of single-target CAR-T treatments [61,62].

Pooled CAR-Ts constitute an amalgamation of two populations of distinct CAR-Ts, each recognizing and engaging a different tumor-associated antigen. For example, a combination of CAR-Ts targeting CD19 and CD123 antigens has emerged as a potent strategy for B-ALL treatment [63]. However, clinical studies have shown that pooled CAR-T represents lower cytokine secretion and cytolysis compared to dual and tandem CAR-Ts [63,64]. 

Dual CAR-T therapy involves the genetic modifications of T cells to express two distinct CARs, thereby increasing the efficiency of the treatment. Clinical trials performed on patients with aggressive B-cell lymphoma have indicated that using CD19/CD22 dual-targeted CAR-Ts not only enhanced the cytotoxic effect against leukemia cells but also reduced the risk of relapse due to the loss of CD19 expression, which is a common resistance mechanism in CAR-T therapy [65]. 

Tandem CAR-Ts are an engineered type of T cells that co-express two distinct antigen-binding domains within a single CAR molecule [2]. For instance, CD123/CLL-1 tandem CAR-Ts demonstrate significantly increased cytotoxicity and cytokine release to AML cells, expressing both CD123 and CLL-1 antigens in comparison to single-target CAR-T therapy [66].

### 5.2. Use of Bispecific Antibodies to Enhance Target Recognition

Bispecific antibodies (BsAbs) have emerged as a considerable strategy to enhance the specificity of target recognition and therapeutic efficiency in the treatment of leukemia. The action of bispecific antibodies consists of the simultaneous targeting of tumor antigens and immune cells, which improves the precision of immune responses and reduces the probability of antigen escape.

Studies indicated that blinatumomab, a bispecific T-cell engager (BiTE) antibody that simultaneously binds with CD19 antigen expressed on the tumor cells and CD3 antigen on T cells, recruits and activates T cells, triggering target-dependent tumor cell killing. As a result, it has been approved for the treatment of B-cell precursor ALL [67,68]. Additionally, MCLA-117 is a bispecific antibody that targets CD3 and CLEC12A, an antigen expressed in AML cells. This BsAb has shown promising results in indicating selective recruitment of T cells to AML cells and triggering their lysis while sparing normal cells [69]. Another Ab, flotetuzumab, is a bispecific CD3-CD123 DART (dual-affinity re-targeting) molecule that targets CD123 antigen, which is frequently overexpressed in AML cells. Clinical trials have indicated that flotetuzumab can induce remission in patients with refractory AML, highlighting its potential as a therapeutic option [70]. Finally, mosunetuzumab is a CD20xCD3 bispecific antibody that targets B-cell malignancies. It has shown efficiency in inducing remission in people with follicular lymphoma who received at least two systemic therapies [71,72].

### 5.3. Genetic Engineering Techniques to Prevent Antigen Loss

Genetic engineering techniques include advanced methods to prevent antigen loss in cancer immunotherapy. Antigen loss is a challenging factor, as tumors frequently downregulate or mutate the target antigens. The point of genetic engineering techniques is modifying genes involved in antigen presentation pathways as well as increasing the recognition and elimination of cancer cells.

CRISPR/Cas9 is a gene editing technique that is used to enhance CAR-T therapies. This technology allows specific modifications in the genome that improve T-cell efficiency and persistence [73]. One approach involves using CRISPR/Cas9 to knock out genes responsible for immune checkpoint proteins or inhibitory receptors on CAR-Ts, such as PD-1 and TGFBR2. By removing these inhibitory signals, CAR-Ts can maintain their activity and continue targeting tumor cells effectively while antigen expression is reduced [74,75]. Additionally, CRISPR/Cas9 can be used to insert multiple CAR constructs into T cells, resulting in recognizing cancer cells expressing different antigens at the same time. This multi-targeting strategy helps mitigate the risk of tumor escape due to antigen loss [76].

Another technique, prime editing (PE), is a highly efficient genome editing technique that allows the insertion of new genetic information into a target site in the case of almost all cell types. In comparison to CRISPR/Cas9, prime editing shows lower off-site activity and does not induce DNA double-strand breaks [77]. As an example, the PE technique can be used to introduce specific mutations into the intrinsic TP53 gene in acute lymphoblastic leukemia cells [78].

TALENs (transcription activator-like effector nucleases) therapy is a gene editing technology composed of nucleases designed to target specific DNA sequences enabling selected gene modifications [79]. Studies have demonstrated that gene editing using this technique for editing CAR-Ts enhances the efficiency of blastic plasmacytoid dendritic cell neoplasm (BPDCN) therapy [80]. 

Thus, the integration of CRISPR/Cas9, prime editing, and TALENs technology into cancer immunotherapy offers promising solutions to overcome antigen loss, increasing the success rate of leukemia treatment.

## 6. Addressing T-Cell Exhaustion: Molecular and Biochemical Approaches

T-cell exhaustion is driven by various factors, including specific epigenetic modifications and inhibitory receptors, such as programmed death-1 (PD-1) or cytotoxic T-lymphocyte-associated protein 4 (CTLA-4, CD152). Strategies eliminating immunosuppressive signals, such as gene editing and engineering switch receptors, can improve CAR-T persistence and antitumor efficacy [81]. Recent advances have identified key factors, such as Tet methylcytosine dioxygenase 2 (TET2), c-Jun transcription factor, basic leucine zipper ATF-like transcription factor (BATF), and Regnase-1, which play crucial roles in regulating exhaustion, offering potential targets for therapeutic intervention [82]. 

One promising strategy to overcome this obstacle is the engineering of CAR-Ts with improved costimulatory domains. These domains are crucial for T-cell proliferation, survival, and effector functions. Incorporating potent co-stimulatory domains, such as 4-1BB (CD137) or CD28, contributes to T-cell proliferation, antigen-specific cytokine secretion, and prolonged antitumor activity [13,83]. Additionally, the application of other costimulatory molecules, including inducible T-Cell co-stimulator (ICOS), OX40 (CD134), CD27, and interleukin-15 (IL15), or by combining multiple co-stimulatory domains, such as in third-generation CAR-Ts, can also be beneficial. The third generation of CAR-T products is characterized by the inclusion of two co-stimulatory domains, with the aim of overcoming the limitations of a single co-stimulatory signal. In both preclinical and clinical trials, third-generation CD19-targeted CAR-Ts have shown improved expansion, survival, and persistence [84,85]. Moreover, modifications to the co-stimulatory domains of CAR-Ts, such as altering one amino acid in the CD28 domain from asparagine to phenylalanine (CD28-YMFM), can enhance the persistence of these cells [86].

Monoclonal antibody-based immune checkpoint blockade (ICB) therapies, mainly including CTLA-4 and PD-1 inhibitors, have shown particular promise in boosting the function of antitumor T lymphocytes [87,88]. High expression of inhibitory receptors, such as PD-1, mucin domain-containing protein 3 (Tim-3), CTLA-4, T-cell immunoreceptor with immunoglobulin and ITIM domains (TIGIT), B and T lymphocyte attenuator (BTLA), 2B4 (CD244), lymphocyte-activation gene 3 (LAG-3), and CD160, has been linked to T-cell exhaustion [89]. Checkpoint inhibitor therapy has been proposed as a potential solution for restoring the activity of exhausted chimeric antigen receptor T cells by targeting pathways that hinder T-cell responses. Although this approach has shown promise, it has been suggested that checkpoint blockade prevents rather than reverses exhaustion [90]. Preclinical studies have shown that combining CAR-T therapy with PD-1 blockade can diminish T-cell exhaustion and increase survival [13,85]. Nevertheless, clinical outcomes vary, presumably because of different cancer types and therapy protocols [85,91]. 

Another potential strategy for sustaining the functionality of CAR-Ts is epigenetic reprogramming. Epigenetic remodeling following CAR-T infusion aims to counteract detrimental epigenetic and transcriptional changes that impact T-cell functions, exhaustion, and tumor infiltration. Strategies such as the use of DNA methyltransferase (DNMT) inhibitors, histone deacetylase (HDAC) inhibitors, and non-coding RNAs (ncRNAs) have enabled CAR-Ts to alleviate exhaustion and enhance therapeutic efficacy. CAR molecules engineered to permit the transient inhibition of CAR expression and tonic signaling or treatment with tyrosine kinase inhibitors (TKI) have shown significant global epigenetic remodeling [92]. 

Dasatinib, a tyrosine kinase inhibitor, can reverse T-cell exhaustion [93]. Targeting DNA methylation pathways, specifically, DNA Methyltransferase 3A (DNMT3A), using pharmacological inhibitors such as decitabine or genetic interventions has been shown to preserve the proliferation and functionality of T cells, allowing for the expression of naive and memory-related genes while downregulating exhaustion-related genes [94,95]. Decitabine (DAC) has been found to reverse the exhaustion of CD123 CAR-Ts and to improve response against AML [96]. Furthermore, the alteration of SUV39H1—a histone 3 lysine 9 methyltransferase that facilitates the formation of heterochromatin—in human CAR-Ts resulted in the reprogramming of the CAR-T phenotype, which maintained its antitumor function [97]. 

Transcription factors, such as nuclear receptor subfamily 4 group A (NR4A) and the thymocyte selection-associated high-mobility group box (TOX) family, are pivotal in the development of T-cell exhaustion, and their upregulation leads to increased inhibitory receptor expression and diminished effector function. Targeting these pathways, potentially using specific miRNAs or CRISPR-Cas9, could reduce exhaustion and enhance the antitumor efficacy of CAR-Ts. Additionally, overexpression of c-Jun can prevent the formation of exhaustion-related complexes, thereby increasing antitumor function and reducing the expression of inhibitory receptors, such as PD-1 and CD39. Moreover, miR-28 and miR-138 can target immune checkpoints and revert exhausted phenotypes, with miR-28 specifically resulting in interleukin-2 (IL-2) and tumor necrosis factor-alpha (TNF-α) secretion in the tumor microenvironment [94]. 

Cellular metabolic pathways play a crucial role in controlling the function and lifespan of T cells. Consequently, modifying the metabolic characteristics of CAR-Ts may be an effective strategy for enhancing their persistence and activity. Specific metabolic pathways can be targeted to promote the development and maintenance of memory T cells owing to differences in metabolism. For instance, glycolysis is essential for effector T-cell functions, whereas memory T cells rely more on oxidative phosphorylation (OXPHOS). Furthermore, adenosine monophosphate-activated protein kinase (AMPK) activation favors memory T-cell formation by suppressing mammalian target of rapamycin (mTOR) signaling, which promotes effector T-cell differentiation [98]. 

The mTOR pathway can be influenced by cytokines such as IL-2 and IL-15. IL-2 stimulates mTOR and enhances glycolysis, leading to the development of effector T-cell phenotypes. In contrast, IL-15 suppresses the activity of mTOR complex 1 (mTORC1), promoting a stem cell memory phenotype in CAR-Ts. Rapamycin inhibits mTORC1, a factor that plays a crucial role in the phosphoinositide 3-kinases (PI3K)-protein kinase B (Akt)-mTOR pathway, contributes to the differentiation of CD8+ T cells into memory T cells. Glycolysis can also be inhibited by the utilization of 2-deoxyglucose (2-DG), which activates AMPK and promotes FAO over glycolysis [98]. 

Another way to promote the metabolic switch to OXPHOS is by elevating the intracellular levels of L-arginine or administering L-arginine supplementation and utilizing compounds such as 6-diazo-5-oxo-L-norleucine (DON) [99]. Interestingly, altering cholesterol levels can also affect T-cell persistence. Specifically, inhibiting acyl-CoA cholesterol acyltransferase 1 (ACAT1) with avasimibe leads to an increase in free cholesterol in T cells by preventing its conversion into cholesteryl esters. Elevated cholesterol enhances T-cell receptor function, promotes larger immunological synapse formation, and boosts CAR-T efficacy in targeting tumors [98]. 

Enhancing the previously mentioned co-stimulatory domains can also affect metabolism. Particularly, CAR-Ts with 4-1BB signaling domains exhibit increased mitochondrial mass, FAO, and biogenesis, resulting in enhanced proliferation, extended persistence, and a memory-like phenotype, compared to CD28 CAR-Ts [100,101,102]. Additionally, immune checkpoint inhibitors (ICIs) such as anti-PD-1 therapies can enhance CAR-T-mitochondrial biogenesis and function, potentially reversing exhaustion and improving cancer treatment outcomes [101]. Another potential strategy to explore is the activation of the interleukin-9 (IL-9)/signal transducer and activator of transcription 3 (STAT3)/fatty acid oxidation pathway. This approach holds promise, as CD8+ Tc9 cells exhibit reduced lipid peroxidation and enhanced resistance to ferroptosis in the tumor microenvironment. As a result, they demonstrate increased persistence and more effective antitumor effects [103]. 

## 7. Combination Therapies and Innovative Approaches

Chimeric antigen receptor T-cell therapy has emerged as a potentially effective approach for treating a range of malignancies, encompassing both hematological cancers and solid tumors [104,105]. However, the efficacy of CAR-T therapy has been impeded by various challenges, including the onset of T-cell exhaustion, which can restrict the long-term effectiveness of this treatment modality [13]. Developing strategies to alleviate T-cell exhaustion and augment the longevity and functionality of CAR-Ts within the tumor microenvironment is pivotal for enhancing the overall efficacy of this therapeutic approach [106].

Small molecule inhibitors have been investigated as a potential approach to enhance the effectiveness of CAR-T therapy by modulating the tumor microenvironment and overcoming resistance factors. Combining CAR-Ts and small molecule inhibitors has demonstrated promising synergistic benefits in preclinical and early clinical studies for leukemia treatment. Small molecule inhibitors can target various pathways that contribute to immune evasion, angiogenesis, and tumor-associated stromal cells, thereby enhancing the trafficking, infiltration, and function of CAR-Ts within the tumor [107]. 

Preclinical investigations have shown that inhibitors directed against the phosphoinositide 3-kinase/protein kinase B/mammalian target of rapamycin (PI3K/AKT/mTOR) signaling pathway can bolster the proliferative capacity, persistence, and anti-neoplastic potency of CAR-Ts in leukemia model systems [108]. Furthermore, inhibitors of the mitogen-activated protein kinase (MAPK) signaling cascade have been observed to augment the cytotoxic capabilities of CAR-Ts against leukemia cells. Additionally, small molecule inhibitors targeting the tumor microenvironment, such as C-X-C chemokine receptor type 4 (CXCR4) antagonists or angiotensin II receptor blockers, have been demonstrated to enhance the infiltration and functional activity of CAR-Ts in the context of leukemia [106,109]. 

The clinical application of these combined therapeutic strategies is actively being investigated. Early-stage clinical trials have reported promising results, with the combination of CAR-Ts and small molecule inhibitors exhibiting enhanced response rates and durability of response in patients with relapsed or refractory leukemia [13]. Various small molecule inhibitors, including those targeting protein kinases, epigenetic modifiers, PI3K/Akt/mTOR signaling, and apoptosis regulators, have demonstrated potential synergistic effects when combined with CAR-T therapy for leukemia [110]. The combined use of CAR-T therapy and small molecule inhibitors exhibits synergistic promise for enhancing the efficacy of leukemia treatment. By mitigating resistance mechanisms and modulating the tumor microenvironment, this integrated therapeutic strategy may provide a more potent and durable solution for patients with leukemia.

Addressing T-cell exhaustion in CAR-T therapy may involve the utilization of immune-modulating agents and cytokine support. The cancer-immunity cycle, which outlines the essential steps for establishing an effective anti-tumor immune response, provides a critical framework for comprehending the role of these interventions [90]. The utilization of immune-modulating agents, including checkpoint inhibitors, can facilitate the overcoming of barriers to T-cell activation and proliferation, thereby augmenting the potency and persistence of chimeric antigen receptor T-cell therapies [111]. Providing cytokines, such as interleukin-15 and interleukin-7, can promote the proliferation, viability, and functional capacity of CAR-Ts, thereby mitigating their premature exhaustion [106]. 

Implementing strategies to enhance the design and administration of CAR-Ts, such as employing dual-antigen recognition or transient RNA-based engineering, can also assist in mitigating the risk of on-target, off-tumor toxicity and improving the targeting of solid tumors [104]. Emerging evidence suggests that immune checkpoint inhibitors, which are monoclonal antibodies, may have a synergistic effect when combined with CAR-T therapy. These checkpoint inhibitors have the potential to overcome the immunosuppressive tumor microenvironment and enhance the proliferative capacity of CAR-Ts, thereby improving the overall efficacy of the combined treatment approach for leukemia [112,113]. Leveraging these approaches and comprehending the mechanisms underlying T-cell exhaustion within the tumor microenvironment enables researchers and clinicians to enhance the long-term efficacy and safety of CAR-T therapy, ultimately improving outcomes for patients with diverse cancers.

The potential of oncolytic viruses to enhance treatment outcomes has been demonstrated across a growing body of research on hematological malignancies [114]. Recent studies have investigated the use of oncolytic viruses, which can selectively proliferate in and destroy tumor cells, as a strategy to augment the functionality of chimeric antigen receptor T cells [115]. Oncolytic viruses can establish a more conducive tumor microenvironment for CAR-Ts by triggering immunogenic tumor cell death, releasing tumor-associated antigens, and enhancing the recruitment and activation of immune effector cells [106]. 

Preclinical studies have demonstrated the promising potential of combining chimeric antigen receptor T-cell therapy with oncolytic viruses. This synergistic approach has resulted in enhanced tumor infiltration, persistence, and anti-tumor efficacy. By addressing the key limitations of CAR-T therapy in solid tumors, the integration of oncolytic viruses may unlock the full therapeutic promise of this innovative treatment modality. CD19-targeted chimeric antigen receptor T-cell therapies (Kymriah and Yescarta) have demonstrated impressive efficacy against B-cell lymphomas and leukemias, while oncolytic virotherapy has elicited notable responses in melanoma (Imlygic and Rigvir) and nasopharyngeal carcinoma (Oncorine) patients. However, the effectiveness of oncolytic viruses as monotherapy is often constrained by the pre-existing and emerging immune response against viral antigens and requires a relatively intact immune system, which is atypical for cancer patients undergoing current anti-tumor treatment regimens [116].

## 8. Case Studies and Preclinical/Clinical Evidence

Studies show that up to 60% of relapses after CD19 CAR-T therapy are characterized by CD19 antigen loss. One of the strategies to overcome this process is targeting multiple antigens. Qin H. and Zah E. et al. suggest that simultaneous targeting of more than one antigen may reduce the likelihood of single-target loss-induced relapse [117,118]. Dai et al. conducted a study where they used autologous bispecific CAR-Ts targeting both CD19 and CD22 on a group of six adult patients with recurrent B-cell acute lymphoblastic leukemia. Their studies confirm that dual targeting is more effective than sequential anti-CD19 CAR-T and anti-CD22 CAR-T infusions in preventing antigen escape. Additionally, they observed in one patient the emergence of both CD19^−^ and ${CD22}^{dim}$ blast cells, which was reported earlier in patients treated sequentially with anti-CD19 CAR-Ts and anti-CD22 CAR-Ts. A reduction in the likelihood of this event can be obtained by using CAR-Ts targeting CD123 or CD38 apart from anti-CD19 and anti-CD22 CAR-Ts [23].

A different way to overcome the process of antigen loss was presented by Chen T. et al. Their modular universal CAR-Ts (MU-CAR-Ts) were able to target various antigens without the need to re-edit autologous or allogeneic T cells [119]. The presence of the Sd/Gv system—extracellular recognition elements derived from Streptococcus dysgalactiae and Gardnerella vaginalis [120]—affects the activation of MU-CAR-Ts and enables redirection of T cells. In this study, generated MU-CAR-Ts were successfully validated for their target recognition and cytotoxicity mediated by VRC01-scFv (single-chain variable fragment) for latent HIV-1-infected cells and CD5-CD30 scFvs for T-cell lymphoma cells. With that, CAR-Ts were able to recognize different antigens without requiring redesign and time-consuming remanufacturing, which might be significant to the optimization of the duration of therapy. Perhaps it will also be possible to use this mechanism in leukemia. 

Another obstacle that prevents the achievement of remission is T-cell exhaustion. Due to no studies or clinical trials specifically addressing T-cell exhaustion in leukemia, we reviewed studies of other hematological cancers using strategies to overcome this problem. Cao et al. used nivolumab, a PD-1 inhibitor, in combination with CD-19 CAR-T in patients with refractory or relapsed B-cell non-Hodgkin lymphoma, achieving remission in around 45% of patients [121].

Jaeger et al. presented a study where attempts were made to stop CAR-T exhaustion by blocking PD-1 with pembrolizumab [122]. Fifteen adult patients with relapsed/refractory diffuse large B-cell lymphoma were treated with pembrolizumab during tisagenlecleucel therapy. Of those, 33.3% had CR, and 16.7% had PR. These results resemble other CAR-T combination trials with checkpoint inhibitors that demonstrate comparable clinical outcomes with CAR-T therapy alone [123,124]. The limited patient sample and short follow-up do not allow for definitive conclusions. However, the use of PD-1 inhibitors might be a way to avoid CAR-T exhaustion in the future. 

## 9. Conclusions, Future Directions, and Research Opportunities

### 9.1. Summary of Key Findings

CAR-T therapy shows promising long-term outcomes in various hematologic malignancies. For instance, in chronic lymphocytic leukemia, two patients have remained in remission for over 10 years post-infusion. The ZUMA-1 trial in relapsed/refractory large B-cell lymphoma reported a 5-year progression-free survival (PFS) rate of 32% and an overall survival (OS) rate of 43%. Non-relapse mortality (NRM), mainly from infections, cardiovascular issues, or rare malignancies, affected 14% of patients. In the ZUMA-7 trial for primary refractory or early relapsed LBCL, second-line axi-cel achieved a 4-year PFS rate of 42% and an OS rate of 55%, with NRM in 14% of cases, primarily due to infections. In relapsed/refractory B-cell acute lymphoblastic leukemia, tisagenlecleucel in the ELIANA trial showed a 3-year relapse-free survival rate of 52% and an OS rate of 63%, with no late treatment-related deaths observed. Trials in mantle cell lymphoma, follicular lymphoma, and multiple myeloma also indicated durable responses, with median PFS rates ranging from 26 to 40 months. However, longer follow-up is needed to assess the potential curative effects of CAR-T therapy in these cancers [61].

### 9.2. Implications for the Future of CAR-T Therapy in Leukemia

CAR-Ts are effective in treating hematological cancers with high efficacy and low toxicity in long-term studies. Durable remissions seen in CD19-targeted CAR-T therapy for B-cell lymphoma demonstrate its potential to cure chemotherapy-resistant cancers. CAR-Ts also facilitate allogeneic HSCT in B-ALL and provide prolonged treatment-free remission in MM. Ongoing research aims to further improve remission durability. Overall, the evolving landscape of CAR-T therapy shows promising improvements in therapeutic responses and expanding treatment options. The main focus of current and future research is on identifying new target antigens and creating novel combinations of existing targets. A key challenge is selecting better preclinical studies to identify promising combinations. Additionally, understanding the mechanisms of antigen loss and finding ways to counteract them is crucial. Addressing inhibitors of T-cell function in the tumor microenvironment can enhance the development of CAR-T products. At present, there are around 470 clinical trials in CAR-T therapy and potentially thousands of combinations to explore [42].

### 9.3. Long-Term Monitoring and Management of CAR-T-Treated Patients to Prevent Relapse

CAR-T therapy, achieving durable remissions in 30–50% of patients, is expanding into new disease indications and earlier treatment lines, leading to a projected increase in long-term survivors. However, this unique population may face complex late complications such as neurotoxicity, prolonged cytopenias, immunosuppression, subsequent malignancies, organ dysfunction, and psychosocial challenges. Prior treatments before CAR-T therapy, such as chemotherapy or stem cell transplants, could influence these late toxicities, necessitating vigilant screening and preventive care practices. A comprehensive understanding of late effects is crucial across different CAR-T products, requiring ongoing research and registry-based monitoring to optimize survivorship care and quality of life [41].

## Figures and Tables

**Table 1:** Comparison of antigen escape and T-cell exhaustion and its influence on CAR-T therapy.

Feature	Car-T Cell Therapy	Antigen Escape	T-Cell Exhaustion
**Definition**	therapy involving the modification of a patient’s T lymphocytes to recognize and destroy cancer cells	the process by which cancer cells change their surface antigens to avoid recognition by the immune system	a loss of function and limited proliferation of T-cells due to prolonged antigen exposure
**Mechanism of Action**	genetic engineering of T lymphocytes to include chimeric antigen receptors (CAR) specific to cancer antigens	alteration or loss of antigens on the surface of cancer cells	expression of inhibitory receptors, epigenetic alterations
**Purpose**	destroying cancer cells by recognizing specific antigens on their surface	avoiding immune response.	not explored
**Methods of Detection**	monitoring therapy effectiveness, analyzing CAR-T cell clonality	gene expression analysis, sequencing, flow cytometry.	gene expression analysis, sequencing, flow cytometry.
**Therapeutic Benefits**	high efficacy in treating certain hematological cancers	limited – mainly associated with avoiding the immune system.	makes therapy less effective
**Challenges and Limitations**	risk of side effects, such as cytokine release syndrome (CRS) and neurotoxicity	rapid adaptation of cancer cells, which leads to disease recurrence	limits the effectiveness of CAR-T therapy, which may lead to disease recurrence or progression
**Examples of Clinical** **Applications**	treatment of leukemia and lymphomas resistant to other therapies	monitoring cancer progression and evaluating the effectiveness of immunotherapies	evaluation of CAR-T therapy effectiveness
**Side Effects**	CRS, neurotoxicity, bone marrow aplasia	may lfead to resistance to immunotherapies	may lead to resistance to immunotherapies
**Research and Development Status**	intensive clinical trials and development of new CAR-T cell generations	research on mechanisms and inhibitors of antigen escape.	research on the mechanism and overcoming it.
**References**	[9,10,11,12,13,14,15]	[30,31,32,33,34,35]	[25,48,49,50,51,52,53,60]

## Data Availability

Most of the original data presented in the study are openly available in online searches.
